# National Variation in Caesarean Section Rates: A Cross Sectional Study in Ireland

**DOI:** 10.1371/journal.pone.0156172

**Published:** 2016-06-09

**Authors:** Sarah-Jo Sinnott, Aoife Brick, Richard Layte, Nathan Cunningham, Michael J. Turner

**Affiliations:** 1 Economic and Social Research Institute, Whitaker Square, Dublin 2, Ireland; 2 Trinity College, Dublin, Ireland; 3 Department of Sociology, Trinity College Dublin, Dublin 2, Ireland; 4 UCD Centre for Human Reproduction, Coombe Women and Infants University Hospital, Dublin 8, Ireland; Stanford University School of Medicine, UNITED STATES

## Abstract

**Objective:**

Internationally, caesarean section (CS) rates are rising. However, mean rates of CS across providers obscure extremes of CS provision. We aimed to quantify variation between all maternity units in Ireland.

**Methods:**

Two national databases, the National Perinatal Reporting System and the Hospital Inpatient Enquiry Scheme, were used to analyse data for all women delivering singleton births weighing ≥500g. We used multilevel models to examine variation between hospitals in Ireland for elective and emergency CS, adjusted for individual level sociodemographic, clinical and organisational variables. Analyses were subsequently stratified for nullipara and multipara with and without prior CS.

**Results:**

The national CS rate was 25.6% (range 18.2% ─ 35.1%). This was highest in multipara with prior CS at 86.1% (range 6.9% ─ 100%). The proportion of variation in CS that was attributable to the hospital of birth was 11.1% (95% CI, 6.0 ─ 19.4) for elective CS and 2.9% (95% CI, 1.4 ─ 5.6) for emergency CS, after adjustment. Stratifying across parity group, variation between hospitals was greatest for multipara with prior CS. Both types of CS were predicted by increasing age, prior history of miscarriage or stillbirth, prior CS, antenatal complications and private model of care.

**Conclusion:**

The proportion of variation attributable to the hospital was higher for elective CS than emergency CS suggesting that variation is more likely influenced by antenatal decision making than intrapartum decision making. Multipara with prior CS were particularly subject to variability, highlighting a need for consensus on appropriate care in this group.

## Introduction

Internationally, the rate of caesarean section (CS) has risen steadily and substantially in recent decades. Across Organisation for Economic Co-operation and Development (OECD) countries, the average rate of CS is now at one in four births, an increase from one in five in 2010.[1]

Although CS is often life-saving for both mother and fetus in instances such as placenta praevia and uterine rupture, this rising rate remains a cause for worry. CS carries almost three times the risk of maternal morbidity and mortality than with vaginal delivery.[2] Other risks include surgical injuries and respiratory distress for the neonate, negative psychosocial implications such as poor perception of birth for the woman and recent evidence demonstrates a negative impact on future fertility. [2–4][5, 6] Rising CS rates also have economic consequences. The World Health Organisation has estimated the global cost of “excess” CS to be $2.3 billion dollars.[7]

Adding to this concern is recent evidence that highlights the wide variation in CS rates both between and within countries. Across 31 European countries in 2010, there was more than a 3 fold variation in CS rates, from 14% at the lower end to 52% at the higher extreme.[8] Similarly, a U.S. study showed that the rate for CS varied from 7% to 70% across 573 hospitals nationally, and this variation was fifteen fold (2% to 36%) for low risk women who should all have had a similar baseline risk. [9] This variation raises three points. First, it highlights the loss of information that can occur when reporting mean rates. The extremes of CS provision are a more useful indicator of obstetric performance and quality, as we have previously highlighted.[10] Second, variation within countries points to a lack of consensus on what CS rates should be. Third, the issue of quality of care is implied given the absence of a standardised approach.

In this study, we used individual level data, from two national databases, on all births in Ireland in 2009 to quantify the amount of variation in CS rates between Irish maternity units. Uniquely, both privately funded and publicly funded births occur on the same wards in Irish hospitals. Thus, our data allow close examination of the association between private funding and CS rates[11, 12], while controlling for factors that may differ between private and public hospitals elsewhere.[12] We hypothesise that clinical and sociodemographic factors, along with organisational factors such as private funding, explain variation in CS rates between hospitals in Ireland.

## Methods

### Data

Data on the woman's marital status, social class, country of birth, along with birthweight, gestational age, obstetric history and parity were sourced from the National Perinatal Reporting System (NPRS), the main source of data on all births (≥500g) in Ireland.[13] A second national data source, the Hospital Inpatient Enquiry (HIPE) scheme, records data on all discharges, and deaths, in all 19 publicly funded maternity units.[14] Diagnosis (one principal and up to 19 additional) codes and procedure (one principal and up to 19 additional) codes are recorded using the Australian Modification of ICD-10 codes (ICD-10-AM) and the *Australian Classification of Health Interventions* (ACHI). Information is also collected on the woman’s age and whether obstetric care was publicly or privately funded.

### Population

The sample comprised all singleton births (live and stillborn) to women discharged from all 19 maternity units in 2009 for whom an NPRS and HIPE record were available; a total of 70,889 births. This represented 96.3% of the total number of singleton births in these maternity units in that year. Homebirths were excluded.

### Models of maternity care

All women in Ireland are entitled to free maternity services, provided from public funds. A proportion, however, choose to finance their care privately. A woman attending publicly typically receives shared antenatal care from her primary care doctor and her chosen hospital. The delivery is attended by a midwife, if uncomplicated. After delivery, the woman is moved to a shared room (usually four or six bedded).

Women who attend privately choose their consultant obstetrician who they see exclusively for the duration of their antenatal care. The consultant, or their nominated consultant colleague, is in attendance for the delivery but the delivery is midwife led if normal. After delivery, a single room (if available) is provided. In the three Dublin hospitals, a third model of care referred to as “semi-private” entitles the woman to a private bed after delivery, but not necessarily consultant provided antenatal care. In our data, semi-private deliveries were coded as private deliveries.

What is unusual about the Irish maternity care system is that both public and private models of care are available on the same labour wards in the 19 maternity units, and both models of care are predominantly midwifery based.

### Variables

The outcome variables in these analyses were elective CS and emergency CS. Elective CS was defined as a CS carried out as a planned procedure before the onset of labour or following the onset of labour, when the decision was made before labour (ICD-10-AM codes 16520–00 and 16520–02). Emergency CS was defined as a CS required because of an emergency situation (e.g., obstructed labour, fetal distress) (ICD-10-AM codes 16520–01 and 16520–03). Explanatory variables included maternal age, social class, maternal country of birth and marital status. Clinical variables were antenatal factors: hypertension, diabetes mellitus, gestational diabetes mellitus (GDM) and (pre)eclampsia. Also included were restricted fetal growth and excessive fetal growth along with placenta praevia, placenta abruption, breech presentation and other forms of malpresentation. Intrapartum difficulties: dystocia; cord prolapse; and fetal distress were not considered in this analysis given the lack of consensus on their definition and their propensity to subjective recording.[15, 16] Due to co-linearity between gestational age and birthweight, we included only a categorical variable for birthweight. Prior stillbirth or miscarriage were included as indicators of obstetric history. Information on model of care, public or private, was included as an organisational variable.

### Statistical Analysis

#### Multilevel regression models

We used multivariable multilevel logistic regression models to assess variation in whether an elective or emergency CS was carried out at the hospital level. From the random intercept models, we calculated a variance partition coefficient (VPC) which describes the between hospital variation as a proportion of all the variation in the dependent variable (CS). We established models for the whole population and established models stratified by parity (nullipara, multipara without prior CS and multipara with prior CS).

#### Plots

We plotted unadjusted proportions of elective and emergency CS per hospital using funnel plots with 95% confidence intervals to graphically display variation between hospitals.[17]

#### Subgroup analyses

To further explore organisational factors, we tested the significance of effect modification by academic hospital status in fully adjusted models with interaction terms between the variable of interest e.g. parity and an indicator for a hospital being an academic hospital. Because effect modification depends on the measurement scale chosen, we modelled interactions using a poisson model to gain estimates of risk.[18] All analyses were carried out using STATA 13.1 for Windows.

#### Hospital Standardised Caesarean Section Rates

We calculated a Hospital Standardised Caesarean Section Rate (HSCSR) for both elective and emergency CS using multilevel models, a methodological advance on prior studies calculating standardised rates for CS. [19, 20] The method we used (method 3 detailed by Mohammed *et al*. [19]) calculated a ratio of the expected outcome of a baseline woman at a particular maternity unit to the outcome expected in the same woman at a baseline hospital, thus reflecting the difference between hospitals for the average woman. A value of one, therefore, indicates no difference in the expected probability of a CS given the attributes of the woman presenting. A value of more than one indicates a higher than expected CS rate, and vice versa.

The control limits represent the area within which all (95%) hospitals should appear assuming differences in rates resulted from random or chance variation. Data points that fall outside the control limits are said to display ‘special-cause variation’, that is, performance diverges significantly from what is expected.

### Ethics

All data were extracted from anonymised administrative sources for secondary analysis. They were obtained and used with the permission of HIPE, the NPRS and the Central Statistics Office (CSO), Ireland *via* a data governance programme led by the CSO and in accordance with the Statistics Act 1993.

## Results

There were 70,889 singleton births in 2009 included in this study. The national average for CS was 25.6% and this ranged from 18.2% to 35.1% between hospitals. 8,270 births (11.7%) were delivered by elective CS and 9,851 births were delivered by emergency CS (13.9%). The population is described in **Table 1.**

**Table 1:** Descriptive characteristics of study population.

	Elective CS		Emergency CS		All other deliveries		Total	
	n = 8270		n = 9851		n = 52768		N = 70889	
**Age (years)**	**n**	**%**	**n**	**%**	**n**	**%**	**n**	**%**
<20	53	0.6	249	2.5	1893	3.6	2195	3.1
20–24	405	4.9	1141	11.6	7089	13.4	8635	12.2
25–29	1329	16.1	2391	24.3	13738	26.0	17458	24.6
30–34	2951	35.7	3404	34.6	17379	32.9	23734	33.5
35–39	2744	33.2	2187	22.2	10831	20.5	15762	22.2
≥40	788	9.5	479	4.9	1838	3.5	3105	4.4
Social Class*								
Professional/managerial	2574	31.1	2992	30.4	14353	27.2	19919	28.1
Clerical	1982	24.0	2530	25.7	12315	23.3	16827	23.7
Skilled/semi-skilled	390	4.7	530	5.4	2785	5.3	3705	5.2
Unskilled	938	11.3	1425	14.5	7225	13.7	9588	13.5
Unemployed	139	1.7	286	2.9	1640	3.1	2065	2.9
Home duties	2041	24.7	1688	17.1	11993	22.7	15722	22.2
Other	190	2.3	382	3.9	2350	4.5	2922	4.1
**Marital Status**								
Married	6333	76.6	6072	61.6	32377	61.4	44782	63.2
Not married	1937	23.4	3779	38.4	20391	38.6	26107	36.8
**Country of Birth**								
Ireland	6813	82.4	7375	74.9	39591	75.0	53779	75.9
UK	197	2.4	241	2.4	1370	2.6	1808	2.6
EU-15**	84	1.0	158	1.6	758	1.4	1000	1.4
EU-27***	459	5.6	921	9.3	6104	11.6	7484	10.6
Africa	301	3.6	447	4.5	1662	3.1	2410	3.4
Asia	255	3.1	493	5.0	2159	4.1	2907	4.1
Other	153	1.9	205	2.1	1040	2.0	1398	2.0
**Funding**								
Private	3534	42.7	2970	30.1	13409	25.4	19913	28.1
Public	4736	57.3	6881	69.9	39359	74.6	50976	71.9
**Parity**								
Nullipara	1577	19.1	6215	63.1	22078	41.8	29870	42.1
Multipara without CS	1259	15.2	2291	23.3	22596	56.1	26146	36.9
Multipara with prior CS	5432	65.7	1343	13.6	1091	2.1	7866	11.1
**History of Stillbirths**								
No previous stillbirth	8128	98.3	9725	98.7	52279	99.1	70132	98.9
Previous stillbirth	142	1.7	126	1.3	489	0.9	757	1.1
**History of Miscarriage**								
No previous miscarriage	5916	71.5	7701	78.2	41275	78.2	54892	77.4
Previous miscarriage	2354	28.5	2150	21.8	11493	21.8	15997	22.6
**Gestational Age at Delivery (weeks)**								
< 33	296	3.6	1109	11.3	1813	3.4	3218	4.5
33–37	7908	95.6	8168	82.9	49023	92.9	65099	91.8
≥38	66	0.8	574	5.8	1932	3.7	2572	3.6
**Birthweight (g)**								
500–1499	27	0.3	263	2.7	279	0.5	569	0.8
1500–2499	216	2.6	752	7.6	1251	2.4	2219	3.1
2500–2999	927	11.2	1220	12.4	5606	10.6	7753	10.9
3000–3499	2874	34.8	2673	27.1	17946	34.0	23493	33.1
3500–3999	2904	35.1	3062	31.1	19132	36.3	25098	35.4
4000–4499	1036	12.5	1480	15.0	7318	13.9	9834	13.9
4500+	286	3.5	401	4.1	1235	2.3	1922	2.7
**Clinical Risk Factors**								
Diabetes mellitus (pre-existing)	63	0.8	68	0.7	92	0.2	223	0.3
Eclampsia or pre-eclampsia	164	2.0	590	6.0	686	1.3	1440	2.0
Gestational diabetes mellitus	289	3.5	286	2.9	819	1.6	1394	2.0
Hypertensive disorder	251	3.0	641	6.5	1596	3.0	2488	3.5
Placenta praevia	195	2.4	159	1.6	16	0.6	370	0.5
Placental abruption	7	0.8	172	1.8	48	0.1	227	0.3
Restricted fetal growth	191	2.3	341	3.5	767	1.5	1299	1.8
Excessive fetal growth	211	2.6	139	1.4	252	0.5	602	0.8
Breech presentation	1566	18.9	709	7.2	158	0.3	2433	3.4
Malpresentation (excl. breech)	460	5.6	248	2.5	152	0.3	860	1.2
Cord prolapse	-	-	76	0.8	21	0.04	97	0.1
Dystocia	-	-	4489	45.6	7311	13.9	11800	16.6
Fetal distress	-	-	4892	49.7	10906	20.7	15798	22.3
Induction of labour	-	-	3251	33.0	14138	26.8	17389	24.5

CS: Caesarean Section

*Socio-economic group is derived from information on maternal occupation, and coded, with minor modifications, using the schema employed by the Central Statistics Office

**EU-15: Belgium, Denmark, France, Germany, Greece, Ireland, Italy, Luxembourg, Netherlands, Portugal, Spain, United Kingdom, Austria, Finland and Sweden.

***EU-27: EU-15 plus Cyprus, Czech Republic, Estonia, Hungary, Latvia, Lithuania, Malta, Poland, Slovakia, Slovenia, Bulgaria and Romania

### Rate of caesarean by parity

**Fig 1:**
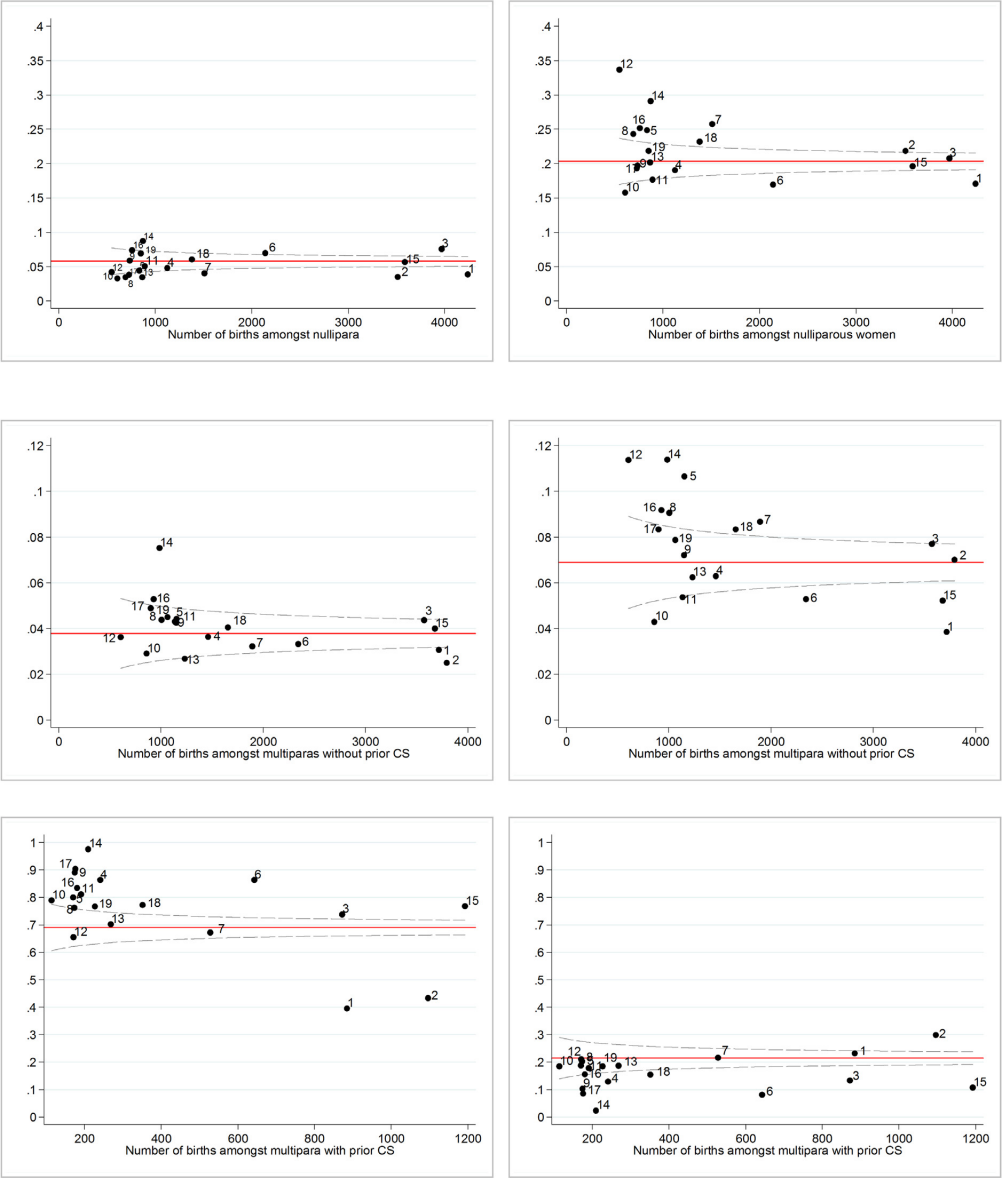
Unadjusted variation between hospitals for elective CS and emergency section. CS- caesarean section. Unadjusted rates. Left panel contains data for elective CS. Right panel contains data for emergency CS.

The unadjusted rates of CS per hospital, and stratified by parity, are demonstrated in **Fig 1.**

### Variation in hospital rates of CS

The proportion of variation in CS that was attributable to the hospital of birth was 11.1% (95% CI, 6.0–19.4) for elective CS and 2.9% (95% CI, 1.4–5.6) for emergency CS.

Adjusted for all other factors, the odds of both elective and emergency CS increased with older age **(Table 2).** Opting for the private model of care increased the odds of both types of CS, as did previous CS and a history of stillbirth or miscarriage. A trend towards decreased odds of elective CS was observed for African and Asian women (OR 0.93, 95% CI 0.73–1.19 and OR 0.77, 95% CI 0.60–0.99). In contrast, both these groups had a higher odds of emergency CS than Irish women (OR 1.79, 95% CI 1.57–2.04 and OR 1.28, 95% CI 1.13–1.44). Breech presentation, other forms of malpresentation and placenta praevia were the strongest predictors of elective CS. Similar clinical indicators predicted emergency CS **(Table 2).**

**Table 2:** Odds of caesarean section across all 19 publicly funded hospitals adjusted by individual level sociodemographic and organisational factors.

	Elective CS			Emergency CS		
	n = 8270			n = 9851		
	OR	95% CI	p	OR	95% CI	p
**Age (ref 30-34yrs) (years)**						
<20	0.40	0.26–0.61	p<0.0001	0.41	0.35–0.48	p<0.0001
20–24	0.57	0.47–0.69	p<0.0001	0.64	0.59–0.7	p<0.0001
25–29	0.74	0.65–0.84	p<0.0001	0.81	0.75–0.86	p<0.0001
35–39	1.16	1.03–1.3	0.011	1.21	1.13–1.3	p<0.0001
≥40	2.53	2.09–3.06	p<0.0001	1.61	1.41–1.83	p<0.0001
Married (ref not married)	1.10	0.98–1.23	0.099	0.90	0.84–0.95	p<0.001
Private (ref public)	2.03	1.82–2.26	p<0.0001	1.16	1.09–1.24	p<0.0001
**Country of birth (ref Ireland)**						
UK	1.05	0.79–1.4	0.718	0.95	0.81–1.12	0.552
EU-15*	0.90	0.6–1.36	0.616	1.00	0.82–1.22	0.987
EU-27**	0.92	0.77–1.1	0.375	0.80	0.74–0.87	p<0.0001
Africa	0.93	0.73–1.19	0.574	1.79	1.57–2.04	p<0.0001
Asia	0.77	0.6–0.99	0.041	1.28	1.13–1.44	p<0.0001
Other	1.12	0.81–1.55	0.486	1.11	0.94–1.32	0.226
**Parity (ref nulliparous)**						
Multipara without CS	0.56	0.49–0.63	p<0.0001	0.18	0.17–0.19	p<0.0001
Multipara with CS***	233.66	205.74–265.36	p<0.0001	4.16	3.78–4.57	p<0.0001
**Obstetric history**						
Previous miscarriage	1.14	1.03–1.27	0.010	1.07	1.01–1.14	0.023
Previous stillbirth	2.26	1.55–3.29	p<0.0001	2.40	1.89–3.05	p<0.0001
**Birthweight (ref = 3500–4000) (g)**						
500–1499	0.07	0.04–0.12	p<0.0001	2.54	2.03–3.17	p<0.0001
1500–2499	1.02	0.76–1.37	0.871	2.15	1.9–2.44	p<0.0001
2500–2999	0.92	0.79–1.08	0.309	0.97	0.89–1.05	0.445
3000–3499	1.05	0.95–1.17	0.347	0.81	0.76–0.86	p<0.0001
4000–4499	0.94	0.81–1.08	0.384	1.43	1.33–1.54	p<0.0001
4500+	1.32	1.02–1.73	0.037	2.32	2.02–2.66	p<0.0001
**Clinical Risk Factors**						
Diabetes mellitus (pre-existing)	8.51	4.68–15.47	p<0.0001	4.00	2.81–5.69	p<0.0001
Eclampsia or pre-eclampsia	3.83	2.9–5.06	p<0.0001	3.46	3.04–3.93	p<0.0001
Gestational diabetes mellitus	2.23	1.7–2.92	p<0.0001	1.68	1.43–1.97	p<0.0001
Hypertensive disorder	1.24	0.96–1.6	0.093	1.74	1.57–1.94	p<0.0001
Placenta praevia***	784.21	437.14–1406.83	p<0.0001	97.15	55.36–170.47	p<0.0001
Placental abruption	1.33	0.39–4.5	0.650	22.11	15.51–31.53	p<0.0001
Restricted fetal growth	4.80	3.6–6.4	p<0.0001	1.41	1.19–1.66	p<0.0001
Excessive fetal growth	15.77	11.91–20.89	p<0.0001	2.57	2.02–3.26	p<0.0001
Breech presentation***	902.01	729.81–1114.85	p<0.0001	40.07	33.08–48.55	p<0.0001
Malpresentation (excl. breech)***	76.31	60.54–96.19	p<0.0001	11.80	9.36–14.87	p<0.0001
Induction of labour				1.38	1.30–1.45	p<0.0001
VPC	11.1% (6% - 19.4%)			2.9% (1.4% - 5.6%)		

CS: Caesarean Section, VPC: Variance Partition Coefficient

*EU-15: Belgium, Denmark, France, Germany, Greece, Ireland, Italy, Luxembourg, Netherlands, Portugal, Spain, United Kingdom, Austria, Finland and Sweden.

**EU-27: EU-15 plus Cyprus, Czech Republic, Estonia, Hungary, Latvia, Lithuania, Malta, Poland, Slovakia, Slovenia, Bulgaria and Romania.

***Note inflated odds ratios for multipara with prior CS, breech, malpresentation and placenta praevia, due to CS being more than 10% prevalent in these particular risk groups. The corresponding risk ratios calculated using poisson regression are in **S1 Table**. [34, 35]

### Variation in hospital rates of CS by parity

The proportion of variation in elective CS that was attributable to the hospital of birth was 5.6% for nullipara, 5.2% for multipara without prior CS and 45% for multipara with prior CS.

Increasing age was predictive of elective CS, but this relationship was not statistically significant across all parity groups. The private model of care was associated with almost twice the odds of elective CS across all parity groups and this was greatest for multipara without prior CS. All other predictors of elective CS by parity are shown in (**S2 Table).**

For emergency CS, the proportion of variation that was attributable to the hospital of birth was 2.5% for nullipara, 4.3% for multipara without prior CS and 29.5% for multipara with prior CS. Older age was predictive of increased odds, although this was not significant for women with prior CS. African and Asian women had a consistently higher odds of emergency CS than Irish women across all parity groups, but this association wasn’t always significant **(S3 Table).**

### Subgroup analyses for academic hospitals

**Fig 2:**
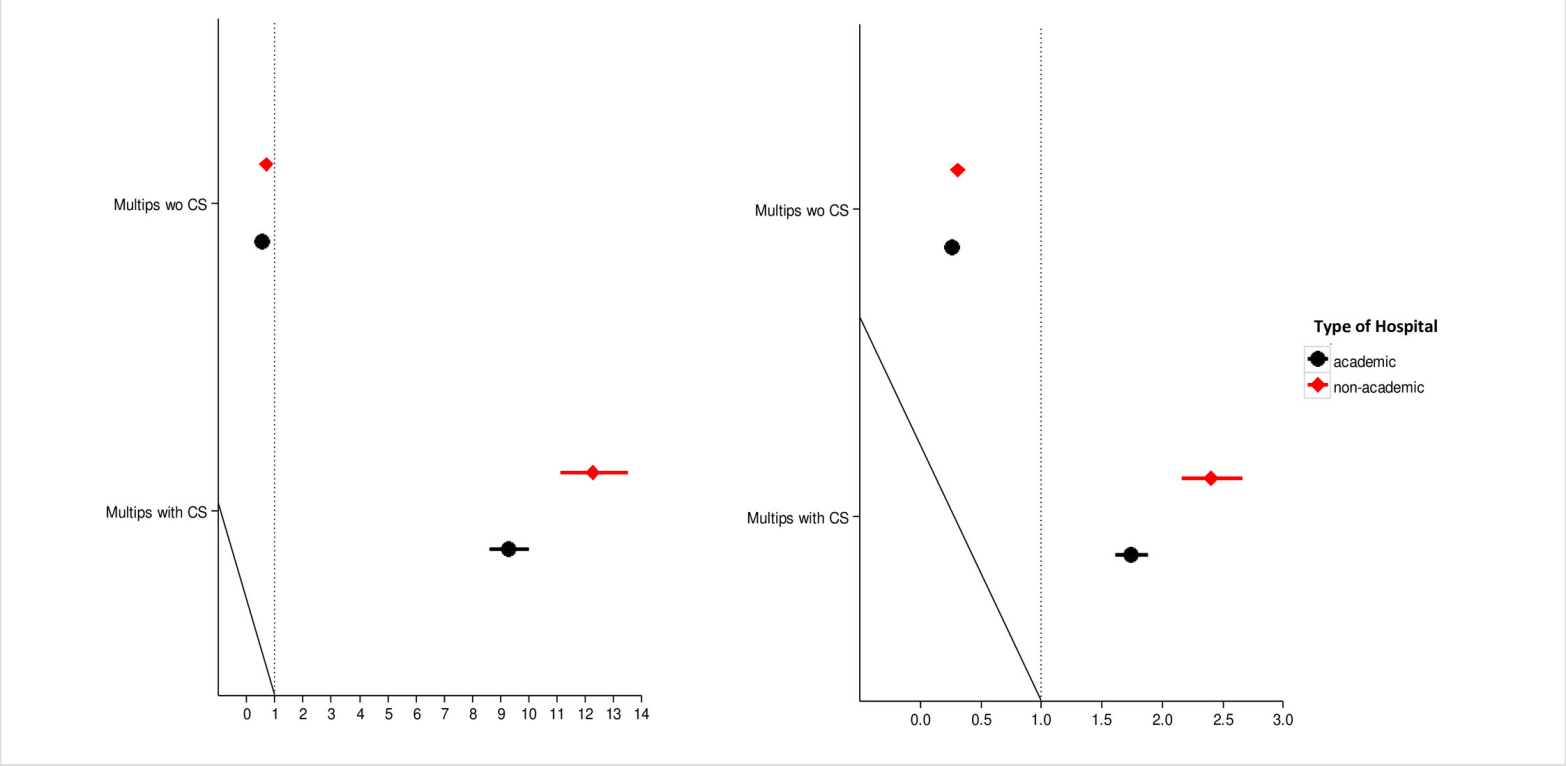
Risk of elective and emergency CS differs for parity groups in academic and non-academic hospitals. CS- caesarean section.

Multipara both with and without prior CS had a decreased risk of both elective and emergency CS in academic hospitals than in other hospitals **(Fig 2)**. Breech presentation was associated with a decreased risk of elective CS in academic hospitals (RR 6.92 *vs* RR 8.12) **(S4 Table).**

### Hospital Standardised Caesarean Section Rates (HSCSRs)

**Fig 3:**
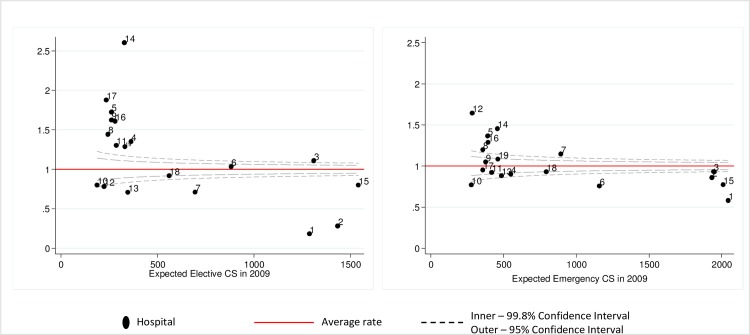
Hospital Standardised Caesarean Section Rates (HSCSRs). CS- caesarean section.

**Fig 3**demonstrates the plotted HSCSRs for elective and emergency CS. For elective CS a number of hospitals did not fall within the control limits suggesting significant differences in factors affecting the risk of elective CS not accounted for by maternal or birth characteristics in our models. The second panel in **Fig 3**demonstrates HSCSRs for emergency CS, which vary to a lesser degree than elective CS.

## Discussion

In this cross-sectional study of more than 70,000 births in Ireland, we adjusted for case-mix, sociodemographic and organisational factors at the individual level, and found that 11% of the variance for elective CS and 3% for emergency CS was due to between hospital variation.

Our results are in line with the existing literature on between hospital variation in CS rates. Several large scale American studies found that after accounting for multiple explanatory causes of CS such as birth factors; hospital capacity; medico-legal claims; socio-economic status; number of beds; teaching hospital status; and rural/urban status unexplained variation persisted. [9, 21] Likewise, in the UK, CS rates varied from 15% to 32% across 146 NHS Trusts in 2008, despite adjusting for various sociodemographic and clinical factors. [22]

Our finding of persistent between hospital variation, despite controlling for detailed maternal and birth factors, suggests that factors such as adherence to evidence based guidelines, professional practises and organisation of care may be contributing to variation and thus may be suitable targets for standardising the CS rate. One American study of variation in CS rates concluded with a policy recommendation of regular audit and feedback to clinicians on their performance, as this has been shown to be an effective hospital policy to reduce CS rates. [23] This policy has recently been tested in a cluster randomised trial with encouraging results.[24]

Our analyses pointed to increasing maternal age being associated with both elective and emergency CS, consistent with previous research.[22, 25] Although socio-economic status is sometimes associated with increased CS rates[26], we did not find a consistent association between social class and CS echoing other previous findings. [27] [28] We found that women born in Africa and Asia had a lower odds of elective CS, but had an increased odds of emergency CS relative to Irish women. Prior studies have found high rates of CS in non-Hispanic black women, but the CS was not defined as elective or emergency. [29] In our stratified analyses, nulliparous African women were at a significantly higher odds than nulliparous Irish women to receive an elective CS, a result that was diluted in the whole population analysis.

We accounted for organisational factors by controlling for models of maternity care and academic hospital practices. The private model of care was associated with increased odds of elective and emergency CS, independent of all other factors, a relationship that is well reported in the literature. [11, 12, 22, 26] This relationship was persistent despite the removal of confounding arising from differences between separate public and private providers occurring in other countries.[12] There was a decreased risk of both elective and emergency CS for multipara with and without prior CS in academic hospitals. This implies that academic hospitals may be better resourced in terms of midwifery and obstetric staff, and may be more willing to supervise a trial of labour after previous section.[30]

Our study was limited by the use of an administrative hospital data collection system, although the quality of coding for CS in HIPE is accepted to be quite high given that its concordance with coding in NPRS was 98% for this study. In addition, HIPE data are inputted by trained and experienced coders, and the system has been rigorously reviewed in the past.[31, 32] However, underreporting of previous CS in some hospitals was identified after the changeover from ICD 9 to ICD 10 codes in 2005. The resulting misclassification bias means that the effect estimates in multipara with prior CS are biased towards the null. It is more difficult to assess the direction of bias in the multipara without CS. A mitigating factor is that our dataset covered more than 96% of births in 2009 with complete national coverage. We also used two complementary data sources to obtain a wide range of clinical and sociodemographic variables at the individual level. The results of our multilevel models were reinforced by our HSCSR analysis. A problem common to most secondary datasets is that some desirable variables for the study question are not always available. Unfortunately, we did not have access to variables for body mass index, smoking or assisted conception all of which are predictors of CS. [26] Nor did we have access to maternal request, although it is has been suggested that maternal request in the absence of a clinical indication may not influence rates. [22]

In conclusion, after accounting for case-mix, sociodemographic and organisational differences, an amount of unexplained variation remained between hospitals. Multipara with prior CS were particularly subject to variable practice, especially for elective CS, drawing attention to a need for consensus on appropriate care in this group. Related to this, multipara (both with and without CS) had a higher odds of CS in non-academic hospitals. Such units may lack the spectrum of resources to respond to medical emergencies, and thus may carry out CS at a lower threshold than in academic hospitals. This finding has practical implications for health service planners and the roll out of Hospital Groups in Ireland policy. [33]

Although we made efforts to control for organisational factors, we believe that unmeasured organisational factors are still at play. We concede that organisational culture is an inherently difficult concept to quantify, but recommended that further research in this particular area may be the best way forward to unravelling complexity. Only when sources of variation are identified and understood can suitable interventions be applied to standardise maternity care and improve quality of care.

## Supporting Information

S1 Table Risk of CS across all 19 publicly funded hospitals adjusted by individual level sociodemographic and organisational factors. (DOCX)

S2 Table Odds of elective CS across all 19 publicly funded hospitals adjusted by individual sociodemographic and organisational factors, by parity. (DOCX)

S3 Table Odds of emergency CS across all 19 publicly funded hospitals adjusted by individual sociodemographic and organisational factors, by parity. (DOCX)

S4 Table Risk of elective and emergency CS differs for parity groups in academic and non-academic hospitals. (DOCX)
