# Supplementary material for: National Variation in Caesarean Section Rates: A Cross Sectional Study in Ireland
Source: PLoS One. 2016 Jun 9;11(6):e0156172. doi: 10.1371/journal.pone.0156172 (PMC4900579; doi:10.1371/journal.pone.0156172)
Supplement: S1 Table — (DOCX) [file pone.0156172.s001.docx]

## S1 Table: Risk of caesarean section across all 19 publicly funded hospitals adjusted by individual level sociodemographic and organisational factors

|  | Elective CS (n=8,270) | | | Emergency CS (n=9,851) | | |
| --- | --- | --- | --- | --- | --- | --- |
|  | **RR** | **95% CI** | **p** | **RR** | **95% CI** | **p** |
| Multipara with CS | 10.30 | 9.69-10.96 | p<0.0001 | 2.06 | 1.93-2.19 | p<0.0001 |
| Placenta praevia | 3.61 | 3.12-4.17 | p<0.0001 | 3.48 | 2.96-4.10 | p<0.0001 |
| Breech presentation | 7.38 | 6.98-7.81 | p<0.0001 | 4.60 | 4.24-4.99 | p<0.0001 |
| Malpresentation (excl. breech) | 2.83 | 2.57-3.13 | p<0.0001 | 3.24 | 2.85-3.69 | p<0.0001 |

All RR are fully adjusted for variables that were included in main model
