# Supplementary material for: National Variation in Caesarean Section Rates: A Cross Sectional Study in Ireland
Source: PLoS One. 2016 Jun 9;11(6):e0156172. doi: 10.1371/journal.pone.0156172 (PMC4900579; doi:10.1371/journal.pone.0156172)
Supplement: S2 Table — (DOCX) [file pone.0156172.s002.docx]

## S2 Table: Odds of elective caesarean section across all 19 publicly funded hospitals adjusted by individual and organisational factors, by parity

|  |  | Nullipara n= 29,870 | | |  | Multipara without CS n= 3,146 | | | Multipara with CS n= 7,866 | | | |
| --- | --- | --- | --- | --- | --- | --- | --- | --- | --- | --- | --- | --- |
|  |  | Elective CS (n=1,577) | | |  | Elective CS (n=1,259) | | | Elective CS (n=5,432) | | | |
|  | n | **OR** | **95% CI** | **p** | **n** | **OR** | **95% CI** | **p** | **n** | **OR** | **95% CI** | **p** |
| Age (ref = 30-34 years) |  |  |  |  |  |  |  |  |  |  |  |  |
| <20 | *42* | 0.46 | 0.26-0.8 | 0.006 | *6* | 1.13 | 0.39-3.26 | 0.827 | *5* | 0.39 | 0.06-2.43 | 0.315 |
| 20-24 | *149* | 0.56 | 0.39-0.8 | 0.001 | *49* | 0.57 | 0.38-0.85 | 0.006 | *206* | 0.78 | 0.55-1.11 | 0.165 |
| 25-29 | *362* | 0.77 | 0.59-0.99 | 0.040 | *198* | 0.72 | 0.57-0.91 | 0.007 | *769* | 0.76 | 0.61-0.95 | 0.018 |
| 35-39 | *329* | 2.17 | 1.69-2.79 | p<0.0001 | *406* | 0.90 | 0.75-1.09 | 0.285 | *2009* | 1.11 | 0.92-1.33 | 0.276 |
| ≥40 | *127* | 11.30 | 8.09-15.79 | p<0.0001 | *122* | 1.48 | 1.11-1.97 | 0.007 | *539* | 1.78 | 1.28-2.48 | 0.001 |
|  |  |  |  |  |  |  |  |  |  |  |  |  |
| Married | *1010* | 1.21 | 0.98-1.5 | 0.076 | *948* | 1.06 | 0.87-1.29 | 0.588 | *4374* | 1.11 | 0.91-1.36 | 0.29 |
|  |  |  |  |  |  |  |  |  |  |  |  |  |
| Private | *664* | 2.36 | 1.89-2.95 | p<0.0001 | *455* | 1.96 | 1.64-2.35 | p<0.0001 | *2414* | 1.90 | 1.58-2.3 | p<0.0001 |
|  |  |  |  |  |  |  |  |  |  |  |  |  |
| Country of Birth (ref = Ireland) |  |  |  |  |  |  |  |  |  |  |  |  |
| UK | *46* | 1.59 | 0.93-2.71 | 0.089 | *42* | 1.15 | 0.75-1.77 | 0.514 | *109* | 0.55 | 0.34-0.9 | 0.016 |
| EU-15 | *38* | 1.10 | 0.59-2.05 | 0.768 | *8* | 0.45 | 0.18-1.14 | 0.091 | *38* | 0.79 | 0.39-1.6 | 0.508 |
| EU-27 | *182* | 1.13 | 0.83-1.54 | 0.435 | *61* | 0.73 | 0.51-1.05 | 0.094 | *216* | 1.01 | 0.72-1.42 | 0.943 |
| Africa | *19* | 2.30 | 1.21-4.36 | 0.011 | *38* | 0.73 | 0.45-1.17 | 0.191 | *244* | 0.93 | 0.68-1.28 | 0.664 |
| Asia | *51* | 0.70 | 0.41-1.22 | 0.210 | *28* | 0.66 | 0.39-1.11 | 0.119 | *176* | 0.87 | 0.6-1.25 | 0.446 |
| Other | *33* | 0.86 | 0.45-1.63 | 0.638 | *19* | 1.28 | 0.72-2.28 | 0.407 | *101* | 1.20 | 0.71-2.03 | 0.498 |
|  |  |  |  |  |  |  |  |  |  |  |  |  |
| Obstetric History |  |  |  |  |  |  |  |  |  |  |  |  |
| Previous miscarriage | *307* | 1.17 | 0.93-1.47 | 0.183 | *393* | 0.93 | 0.78-1.1 | 0.389 | *1653* | 1.53 | 1.28-1.81 | p<0.0001 |
| Previous stillbirths |  | 1.00 | 0-0 |  | *35* | 2.25 | 1.45-3.5 | 0.000 | *105* | 2.77 | 1.39-5.51 | 0.004 |
|  |  |  |  |  |  |  |  |  |  |  |  |  |
| Birthweight (ref= 3500 – 3999g) | | | | |  |  |  |  |  |  |  |  |
| 500-1499 | ***16*** | 0.14 | 0.06-0.34 | p<0.0001 | *8* | 0.06 | 0.02-0.14 | p<0.0001 | *3* | 0.02 | 0-0.14 | p<0.0001 |
| 1500-2499 | *82* | 2.61 | 1.73-3.94 | p<0.0001 | *49* | 0.84 | 0.49-1.41 | 0.504 | *85* | 0.37 | 0.22-0.62 | 0.000 |
| 2500-2999 | *226* | 1.23 | 0.91-1.67 | 0.184 | *147* | 0.88 | 0.65-1.18 | 0.390 | *554* | 0.84 | 0.65-1.09 | 0.188 |
| 3000-3499 | *612* | 1.17 | 0.93-1.46 | 0.178 | *422* | 1.04 | 0.87-1.25 | 0.672 | *1840* | 1.01 | 0.84-1.21 | 0.906 |
| 4000-4499 | *143* | 1.19 | 0.86-1.64 | 0.307 | *134* | 0.76 | 0.6-0.96 | 0.022 | *759* | 1.01 | 0.79-1.29 | 0.937 |
| 4500+ | *51* | 2.78 | 1.62-4.77 | p<0.001 | *51* | 1.26 | 0.86-1.86 | 0.237 | *184* | 0.84 | 0.53-1.31 | 0.440 |
|  |  |  |  |  |  |  |  |  |  |  |  |  |
| Clinical Risk Factors |  |  |  |  |  |  |  |  |  |  |  |  |
| Diabetes mellitus (pre-existing) | *13* | 16.42 | 5.9-45.73 | p<0.0001 | *9* | 4.30 | 1.69-10.94 | 0.002 | *41* | 37.59 | 3.07-460.39 | 0.005 |
| Eclampsia or pre-eclampsia | *61* | 3.96 | 2.72-5.77 | p<0.0001 | *29* | 5.08 | 3.17-8.14 | p<0.0001 | *74* | 2.87 | 1.19-6.95 | 0.019 |
| Gestational diabetes mellitus | *45* | 2.70 | 1.62-4.5 | p<0.001 | *44* | 1.92 | 1.24-2.95 | 0.003 | *200* | 2.25 | 1.42-3.56 | p<0.001 |
| Hypertensive disorder | *73* | 1.24 | 0.84-1.83 | 0.281 | *40* | 1.12 | 0.71-1.78 | 0.623 | *138* | 1.35 | 0.81-2.27 | 0.250 |
| Placenta praevia | *58* | 401.86 | 169.65-951.92 | p<0.0001 | *100* | 812.92 | 372.87-1772.31 | p<0.0001 | *37* | na | 0-0 | 0.998 |
| Restricted fetal growth | *85* | 4.68 | 3.13-7 | p<0.0001 | *42* | 3.82 | 2.4-6.07 | p<0.0001 | *64* | 3.35 | 1.47-7.63 | 0.004 |
| Excessive fetal growth | *82* | 31.37 | 20.01-49.19 | p<0.0001 | *40* | 8.16 | 5.31-12.54 | p<0.0001 | *89* | na | 0-0 | 0.997 |
| Breech presentation | *875* | 1971.3 | 1389.06-2797.59 | p<0.0001 | *480* | 379.79 | 290.89-495.86 | p<0.0001 | *211* | 56.02 | 11.28-278.26 | p<0.0001 |
| Malpresentation (excl. breech) | *164* | 160.65 | 106.56-242.21 | p<0.0001 | *129* | 59.01 | 43.83-79.45 | p<0.0001 | *167* | 33.20 | 4.55-241.94 | p<0.001 |
|  |  |  |  |  |  |  |  |  |  |  |  |  |
| VPC |  | 5.6% (2.7-11.4) | | |  | 5.2% (2.5-10.6) | | |  | 45.0% (28.0-63.1) | | |

VPC: Variance Partition Coefficient, CS: Caesarean Section
