# Supplementary material for: National Variation in Caesarean Section Rates: A Cross Sectional Study in Ireland
Source: PLoS One. 2016 Jun 9;11(6):e0156172. doi: 10.1371/journal.pone.0156172 (PMC4900579; doi:10.1371/journal.pone.0156172)
Supplement: S3 Table — (DOCX) [file pone.0156172.s003.docx]

## S3 Table: Odds of emergency caesarean section across all 19 publicly funded hospitals adjusted by individual and organisational factors, by parity

|  | Nullipara n= 29,870 | | | | | Multipara without CS n= 3,146 | | | | | | | | Multipara with CS n= 7,866 | | | | |
| --- | --- | --- | --- | --- | --- | --- | --- | --- | --- | --- | --- | --- | --- | --- | --- | --- | --- | --- |
|  | Emergency CS (n=9,851) | | | | | Emergency CS (n=2,291) | | | | | | | | Emergency CS (n=1,343) | | | | |
|  |  | |  |  |  |  | |  |  | | |  | |  | |  |  |  |
|  | ***n*** | | **OR** | **95% CI** | **p** | **n** | | **OR** | **95% CI** | | | **p** | | ***n*** | | **OR** | **95% CI** | **p** |
| Age (ref = 30-34 Years) | | | | | | | | | | | | | | | | | | |
| <20 | *238* | | 0.42 | 0.36-0.5 | p<0.0001 | *7* | | 0.51 | 0.23-1.16 | | | 0.108 | |  |  | |  |  |
| 20-24 | *914* | | 0.63 | 0.56-0.69 | p<0.0001 | *146* | | 0.84 | 0.68-1.03 | | | 0.094 | | *81* | 0.85 | | 0.58-1.24 | 0.396 |
| 25-29 | *1692* | | 0.78 | 0.71-0.84 | p<0.0001 | *464* | | 0.95 | 0.83-1.09 | | | 0.438 | | *234* | 0.83 | | 0.63-1.08 | 0.163 |
| 35-39 | *1047* | | 1.41 | 1.29-1.55 | p<0.0001 | *717* | | 1.17 | 1.03-1.32 | | | 0.012 | | *422* | 0.90 | | 0.72-1.12 | 0.362 |
| ≥40 | *184* | | 1.70 | 1.38-2.1 | p<0.0001 | *210* | | 1.84 | 1.53-2.22 | | | p<0.0001 | | *85* | 0.87 | | 0.58-1.31 | 0.516 |
|  |  | |  |  |  |  | |  |  | | |  | |  |  | |  |  |
| Married | *3507* | | 0.94 | 0.88-1.01 | 0.097 | *1560* | | 0.82 | 0.73-0.92 | | | 0.001 | | *1005* | 0.89 | | 0.71-1.12 | 0.338 |
|  |  | |  |  |  |  | |  |  | | |  | |  |  | |  |  |
| Private | *1890* | | 1.08 | 1-1.17 | 0.050 | *602* | | 1.30 | 1.15-1.47 | | | p<0.0001 | | *478* | 1.56 | | 1.24-1.96 | p<0.001 |
|  |  | |  |  |  |  | |  |  | | |  | |  |  | |  |  |
| Country of Birth (ref = Ireland) | | | | | | | | | | | | | | | | | |  |
| UK | *122* | | 0.87 | 0.7-1.09 | 0.222 | *73* | | 1.12 | 0.85-1.47 | | | 0.433 | | *46* | 0.91 | | 0.52-1.57 | 0.727 |
| EU-15 | *117* | | 0.95 | 0.76-1.19 | 0.661 | *24* | | 0.92 | 0.57-1.49 | | | 0.745 | | *17* | 1.83 | | 0.85-3.92 | 0.122 |
| EU-27 | *704* | | 0.74 | 0.67-0.82 | p<0.0001 | *157* | | 1.13 | 0.93-1.37 | | | 0.209 | | *60* | 0.96 | | 0.64-1.46 | 0.857 |
| Africa | *154* | | 1.77 | 1.44-2.18 | p<0.0001 | *173* | | 2.07 | 1.7-2.53 | | | p<0.0001 | | *119* | 1.74 | | 1.22-2.47 | 0.002 |
| Asia | *304* | | 1.21 | 1.04-1.4 | 0.014 | *90* | | 1.26 | 0.98-1.64 | | | 0.075 | | *99* | 1.79 | | 1.22-2.63 | 0.003 |
| Other | *136* | | 1.02 | 0.83-1.25 | 0.868 | *44* | | 1.45 | 1.02-2.05 | | | 0.037 | | *25* | 1.08 | | 0.57-2.06 | 0.814 |
|  |  | |  |  |  |  | |  |  | | |  | |  |  | |  |  |
| Obstetric History | | | | | | | | | | | | | | | | | |  |
| One or more previous miscarriage | *1052* | | 1.13 | 1.04-1.23 | 0.004 | *672* | | 0.94 | 0.84-1.05 | | | 0.261 | | *424* | 1.34 | | 1.1-1.64 | 0.004 |
| One or more previous stillbirths |  | | 1.00 | 0-0 |  | *94* | | 2.48 | 1.91-3.21 | | | p<0.0001 | | *30* | 1.61 | | 0.74-3.51 | 0.231 |
|  |  | |  |  |  |  | |  |  | | |  | |  |  | |  |  |
| Birthweight (ref = 3500-3999g) | | | | | | | | | | | | | | | | | | |
| 500-2499 | *149* | | 2.38 | 1.8-3.15 | p<0.0001 | *86* | | 3.05 | 2.06-4.53 | | p<0.0001 | | | *28* | 2.12 | | 0.81-5.52 | 0.125 |
| 2500-2999 | *388* | | 1.55 | 1.32-1.83 | p<0.0001 | *266* | | 4.49 | 3.63-5.55 | | p<0.0001 | | | *97* | 1.47 | | 0.92-2.35 | 0.109 |
| 3000-3499 | *736* | | 0.82 | 0.74-0.91 | p<0.001 | *287* | | 1.48 | 1.25-1.75 | | p<0.0001 | | | *197* | 1.07 | | 0.8-1.43 | 0.649 |
| 4000-4499 | *1753* | | 0.77 | 0.71-0.83 | p<0.0001 | *574* | | 0.99 | 0.88-1.12 | | 0.904 | | | *346* | 0.73 | | 0.59-0.92 | 0.007 |
| 4500+ | *980* | | 1.68 | 1.53-1.85 | p<0.0001 | *321* | | 1.21 | 1.04-1.39 | | 0.012 | | | *178* | 0.93 | | 0.7-1.24 | 0.613 |
|  |  | |  |  |  |  | |  |  | |  | | |  |  | |  |  |
| Clinical Risk Factors | | | | | | | | | | | | | | | | | | |
| Diabetes mellitus (pre-existing) | *45* | 4.78 | | 2.95-7.76 | p<0.0001 | *19* | 3.71 | | | 2.05-6.73 | | | p<0.0001 |  | | na |  |  |
| Eclampsia or pre-eclampsia | *446* | 3.09 | | 2.66-3.57 | p<0.0001 | *107* | 5.54 | | | 4.17-7.36 | | | p<0.0001 | *37* | 3.78 | | 1.59-9.01 | 0.003 |
| Gestational diabetes mellitus | *149* | 1.64 | | 1.32-2.04 | p<0.0001 | *83* | 1.91 | | | 1.46-2.51 | | | p<0.0001 | *54* | 1.62 | | 0.94-2.76 | 0.080 |
| Hypertensive disorder | *453* | 1.59 | | 1.4-1.81 | p<0.0001 | *109* | 1.97 | | | 1.55-2.5 | | | p<0.0001 | *78* | 3.08 | | 1.85-5.15 | p<0.0001 |
| Placenta praevia | *43* | 21.97 | | 9.65-50.03 | p<0.0001 | *86* | 133.58 | | | 63.09-282.85 | | | p<0.0001 | *30* | na | |  |  |
| Placental abruption | *73* | 9.51 | | 5.88-15.38 | p<0.0001 | *85* | 42.42 | | | 25.16-71.51 | | | p<0.0001 | *14* | 3.35 | | 0.89-12.7 | 0.075 |
| Restricted fetal growth | *236* | 1.33 | | 1.1-1.62 | 0.004 | *82* | 1.82 | | | 1.34-2.48 | | | p<0.001 | *23* | 1.41 | | 0.59-3.38 | 0.439 |
| Excessive fetal Growth | *90* | 2.71 | | 1.94-3.78 | p<0.0001 | *30* | 2.61 | | | 1.7-3.99 | | | p<0.0001 | *19* | na | |  |  |
| Breech presentation | *361* | 35.91 | | 26.52-48.61 | p<0.0001 | *290* | 38.87 | | | 30.32-49.84 | | | p<0.0001 | *58* | 25.67 | | 6.16-106.9 | p<0.0001 |
| Malpresentation (excl. breech) | *101* | 8.65 | | 5.88-12.74 | p<0.0001 | *109* | 15.40 | | | 11.51-20.61 | | | p<0.0001 | *38* | 44.80 | | 6.06-331.24 | p<0.001 |
| Induction of labour | *2626* | 1.71 | | 1.60-1.82 | p<0.0001 | *500* | 0.78 | | | 0.69-0.88 | | p<0.0001 | | *125* | 0.72 | | 0.55-0.94 | 0.017 |
|  |  | | | | |  | | | | | | | |  | | | | |
| VPC | 2.5% (1.2%-4.9%)  *VPC: Variance Partition Coefficient, CS: Caesarean Section* | | | | | 4.3% (2.2-8.1) | | | | | | | | 29.5%(15.9-48.2) | | | | |

VPC: Variance Partition Coefficient, CS: Caesarean Section
