# Supplementary material for: National Variation in Caesarean Section Rates: A Cross Sectional Study in Ireland
Source: PLoS One. 2016 Jun 9;11(6):e0156172. doi: 10.1371/journal.pone.0156172 (PMC4900579; doi:10.1371/journal.pone.0156172)
Supplement: S4 Table — (DOCX) [file pone.0156172.s004.docx]

## S4 Table: Risk of elective and emergency caesarean section differs for parity groups in academic and non-academic hospitals

| Subgroup effects for academic hospitals | | | | |
| --- | --- | --- | --- | --- |
|  | **Academic Hospitals** | | **Non-academic Hospitals** | |
| Elective Sections | **RR** | **95% CI** | **RR** | **95% CI** |
|  |  |  |  |  |
| Multipara without CS | 0.55 | 0.50-0.61 | 0.69 | 0.61-0.77 |
| Multipara with CS | 9.29 | 8.62-10.02 | 12.26 | 11.13-13.52 |
| Breech presentation | 6.93 | 6.44-7.45 | 8.12 | 7.43-8.88 |
| Malpresentation (excl. breech) | 1.22 | 1.00-1.49 | 3.22 | 2.74-3.77 |
|  |  |  |  |  |
| Emergency Sections |  |  |  |  |
| Multipara without CS | 0.26 | 0.24-0.28 | 0.31 | 0.29-0.33 |
| Multipara with CS | 1.74 | 1.61-1.88 | 2.40 | 2.16-2.66 |
